# CT Perfusion: Technical Developments and Current and Future Applications

**DOI:** 10.1155/2015/397521

**Published:** 2015-01-28

**Authors:** Maria Antonietta Mazzei, Lorenzo Preda, Alessandro Cianfoni, Luca Volterrani

**Affiliations:** ^1^Department of Medical, Surgical and Neuro Sciences, Diagnostic Imaging, University of Siena, Viale Bracci 10, 53100 Siena, Italy; ^2^Division of Radiology, European Institute of Oncology, University of Milan, Via Ripamonti 435, 20141 Milan, Italy; ^3^Department of Neuroradiology, Medical University of South Carolina, Charleston, SC 29425, USA; ^4^Department of Neuroradiology, Neurocenter of Italian Switzerland, 6903 Lugano, Switzerland

The official history of Computed Tomography perfusion (CTp) began in 1979 when Heinz and his colleagues published their paper [1].

From that date, a limited number of experiences have been carried out to achieve the technique that is now becoming more familiar thanks to the availability of commercial CTp software platforms that are integrated into today's clinical reporting workstations, allowing a rapid analysis and processing of dynamic data sets. From a technical point of view, CTp requires a rapid intravenous injection of an iodinated contrast medium (CM) bolus and sequential imaging to simultaneously monitor changes in the CM concentration as a function of time, both in the tissue of interest and in the vessel that is used as an input function. CTp is thus able to determine, through mathematical models and dedicated software, the perfusion parameters of a given tissue, such as the blood flow (BF), the blood volume (BV), the mean transit time (MTT), and the capillary permeability surface (PS). In particular, PS is considered a functional CT surrogate marker of neoplastic angiogenesis, focusing on the interest of the use of CTp in oncologic imaging [2]. Today, CTp could routinely offer functional imaging information, as an adjunct to a conventional or morphologic CT examination. In particular, it is a widely applied technique in the evaluation of acute ischemic stroke patients and to investigate other brain diseases, including tumours. It can also be used as an aid to distinguish benign and malignant lesions in body imaging and above all to monitor the treatment response in oncologic patients. This has subsequently lead to some authors affirming that CTp is a more sensitive image biomarker than RECIST and tumour density for monitoring early antiangiogenic treatment effects as well as in predicting prognosis and progression-free survival at the end of treatment. However, despite these possibilities, CTp is still considered a niche technique because of some issues. Firstly, the lack of awareness between radiologists, in fact despite the diffusion of commercial CTp software platforms, CTp has not been routinely utilised in clinical practice yet. It also suffers from some limitations due to the high radiation dose delivered to patients, the need for CM injection, and the lack of reproducibility of CTp data obtained from different software packages used and also between the different upgrades of the same commercial software [3]. The dose exposure to the patient is strictly related to the acquisition time required for the dynamic scanning of the volume being analysed. However, a lot of possibilities for reducing the dose exposure to the patient are available today, for example, the axial acquisition opposed to the cine acquisition technique, the combination between CTp and iterative reconstruction techniques, and the possibility to obtain reproducible CTp measurements using a short time acquisition protocol with the CTp deconvolution method. Therefore, if the dose exposure question can be overtaken today, the main problem remains the lack of a standardised method for CTp analysis. Several algorithms have been developed, applying different kinetic models. The algorithms of these software packages were categorised into two groups on the basis of the applied model and the effect of the delay of the bolus tracer: delay-sensitive and delay-insensitive. In particular, the two main models used by different software packages for CTp analysis derive the Time-Density Curves (TDC) and consequently the CTp parameters of a given tissue by using a graphic analysis of a two-compartment model, the so-called Patlak plot, or a deconvolution technique based on the time invariant linear compartmental model, that uses arterial and tissue TDCs to calculate the residual impulse response function (IRF), a theoretic curve obtained assuming that the CM is not diffusible. Recent studies showed that intervendor differences constituted the primary cause for the variability in CTp analysis; moreover, there is also a lack of reproducibility of CTp values obtained from different upgrades of the same commercial software. In fact, even if upgrades of the same commercial software frequently improve reliability and performance, such upgrades may significantly alter the derived CTp parameter values with a potential clinical impact, in particular, in oncologic imaging. Because of this variability, CTp summary maps should be interpreted with care and future studies on this topic should be focused on the standardisation of CTp analysis algorithms in order to obtain reproducible and comparable results across different institutions and different software packages. In this special issue, we collected articles focusing on some interesting topics in CTp imaging. In particular, new aspects of investigation of CTp in oncologic imaging are discussed in three articles: “Perfusion in the Tissue Surrounding Pancreatic Cancer and the Patient's Prognosis,” “CT Perfusion in the Characterisation of Renal Lesions: An Added Value to Multiphasic CT,” and “Role of CT Perfusion in Monitoring and Prediction of Response to Therapy of Head and Neck Squamous Cell Carcinoma,” whereas technical aspects regarding the possibilities of reducing the dose exposure to the patient and protocol optimisation are discussed in the “Reduced Time CT Perfusion Acquisitions Are Sufficient to Measure the Permeability Surface Area Product with a Deconvolution Method” and “Total Bolus Extraction Method Improves Arterial Image Quality in Dynamic CTAs Derived from Whole-Brain CTP Data,” respectively. The contributions of this special issue could stimulate the spread of CTp imaging in daily practice, pinpoint new or extended applications of this technique, and share some strategies to optimise CTp protocol also in reducing the radiation dose to the patient.

*Maria Antonietta Mazzei* *Lorenzo Preda* *Alessandro Cianfoni* *Luca Volterrani*
